# CD80 Expression Correlates with IL-6 Production in THP-1-Like Macrophages Costimulated with LPS and Dialyzable Leukocyte Extract (Transferon®)

**DOI:** 10.1155/2019/2198508

**Published:** 2019-04-10

**Authors:** Alexis P. Jiménez-Uribe, Hugo Valencia-Martínez, Gregorio Carballo-Uicab, Luis Vallejo-Castillo, Emilio Medina-Rivero, Rommel Chacón-Salinas, Lenin Pavón, Marco A. Velasco-Velázquez, Gabriela Mellado-Sánchez, Sergio Estrada-Parra, Sonia M. Pérez-Tapia

**Affiliations:** ^1^Unidad de Desarrollo e Investigación en Bioprocesos (UDIBI), Escuela Nacional de Ciencias Biológicas, Instituto Politécnico Nacional, Ciudad de México 11340, Mexico; ^2^Departamento de Farmacología, Centro de Investigación y de Estudios Avanzados (CINVESTAV) del IPN, Ciudad de México 07360, Mexico; ^3^Departamento de Inmunología, Escuela Nacional de Ciencias Biológicas, Instituto Politécnico Nacional, Ciudad de México 11340, Mexico; ^4^Laboratorio de Psicoinmunología, Dirección de Investigaciones en Neurociencias del Instituto Nacional de Psiquiatría Ramón de la Fuente, Ciudad de México 14370, Mexico; ^5^Departamento de Farmacología y Unidad Periférica de Investigación en Biomedicina Traslacional (CMN 20 de Noviembre, ISSSTE), Facultad de Medicina, Universidad Nacional Autónoma de México, Ciudad Universitaria, Ciudad de México 04510, Mexico

## Abstract

Transferon® is a complex drug based on a mixture of low molecular weight peptides. This biotherapeutic is employed as a coadjuvant in clinical trials of several diseases, including viral infections and allergies. Given that macrophages play key roles in pathogen recognition, phagocytosis, processing, and antigen presentation, we evaluated the effect of Transferon® on phenotype and function of macrophage-like cells derived from THP-1 monocytes. We determined the surface expression of CD80 and CD86 by flow cytometry and IL-1*β*, TNF-*α*, and IL-6 levels by ELISA. Transferon® alone did not alter the steady state of PMA-differentiated macrophage-like THP-1 cells. On the contrary, simultaneous stimulation of cells with Transferon® and LPS elicited a significant increase in CD80 (*P* ≤ 0.001) and CD86 (*P* ≤ 0.001) expression, as well as in IL-6 production (*P* ≤ 0.05) compared to the LPS control. CD80 expression and IL-6 production exhibited a positive correlation (*r* = 0.6, *P* ≤ 0.05) in cells exposed to Transferon® and LPS. Our results suggest that the administration of Transferon® induces the expression of costimulatory molecules and the secretion of cytokines in LPS-activated macrophages. Further studies are necessary to determine the implication of these findings in the therapeutic properties of Transferon®.

## 1. Introduction

Transferon® is a human dialyzable leucocyte extract (DLE), and its active pharmaceutical ingredient is a complex mixture of low molecular weight peptides with immunomodulatory properties. This complex biological drug has been used in several clinical trials in immune-related diseases like herpes simplex infection, atopic dermatitis, asthma, and allergy [1–7]. At the cellular level, DLEs activate TLR signaling in monocytes [8], NF-*κ*B and cAMP in endothelial cells, and modify cytokine production in candida and herpes infection [3, 9]. Another dialyzable leukocyte extracts, such as bovine source (bDLEs), decrease nitric oxide and TNF-*α* production in LPS-stimulated murine peritoneal macrophages [10] and induce macrophage differentiation to M2 alternative profile [11]. Despite this evidence and by its complex composition, DLE could have diverse effects at the cellular level.

The mononuclear phagocytic system, which involves the differentiation of hematopoietic cells into blood monocytes and tissue macrophages, is an essential part of the innate immune system [12] since it performs several key functions, including phagocytosis, production of cell-activating cytokines, tissue remodeling, antigen presenting through MHC-II, and expression of costimulatory molecules. Altogether, these functions favor an appropriate adaptive immune response [13]. For example, during infections, macrophages can be activated by pathogen components, such as lipopolysaccharide (LPS) from Gram-negative bacteria, which is recognized by Toll-like receptor 4 (TLR-4) [14]. In consequence, macrophages enhance their cytokine production and antigen-presenting cell (APC) capabilities [15].

Macrophages internalize and process antigens to present them to lymphocyte T-cell through MHC-II molecules [16]; however, other costimulatory signals are required to induce a full T-cell activation. CD80 and CD86 are the main costimulatory molecules expressed on the APC surface. CD80 and CD86 bind to CD28 or CD152 to promote or restrict T-cell activation depending on time and signal strength [17]. Once T-cells are activated, certain cytokines will define their polarization profile.

IL-6 is a cytokine involved in several immune functions; it could be produced by APC to promote polarization to the anti-inflammatory Th2 profile [18, 19] and induce a Th17 profile during priming of T-cells [20]. Furthermore, IL-6 also influences the acquisition of an M2 phenotype in macrophages [21].

The human monocytic cell line THP-1 has been extensively used for an *in vitro* study of the mononuclear phagocytic system. THP-1 cells can be easily differentiated into macrophage-like cells with increased adherence, loss of proliferative activity, and CD14^+^/CD11b^+^ phenotype [22]. In this work, we aimed to evaluate the effect of leukocyte dialyzable extract, Transferon®, on CD80 and CD86 surface expression as well as their relationship with IL-6 production in immunomodulatory macrophage-like THP1 cells.

## 2. Material and Methods

### 2.1. THP-1 Culture and Differentiation

THP-1 monocytes were obtained from ATCC® (Manassas, VA, USA). Cells were maintained in RPMI 1640 medium (ATCC®) supplemented with 10% heat-inactivated fetal bovine serum (FBS) (GIBCO®, Waltham, Massachusetts, USA) and 0.05 mM 2-mercaptoethanol (Sigma®, St. Louis Missouri, USA) during 5 days in a humidified incubator at 37°C with 5% of CO_2_. THP-1 monocytes were differentiated to macrophage-like cells as previously reported [23]. Briefly, cells were exposed to 50 ng/mL phorbol myristate acetate (PMA) (Sigma®) during 24 h and then incubated for additional 24 h in the absence of PMA before further treatment. Macrophage-like THP1 cells were harvested by washing them with cold PBS (Gibco®) with 1% FBS and then exposed to cold 25 mM EDTA (Invitrogen®, AM9260G) for 10 min in ice. Cells were gently removed, suspended, and washed with RPMI 1640 medium containing 10% FBS. Cells were counted, and the viability was assessed using trypan blue (Sigma®, T8154-100ML). In all experiments, we used microscopy to confirm that PMA treatment induced the expected morphological changes; we also evaluated CD14 and CD11 expression by flow cytometry using anti-CD14 (BD Biosciences, La Jolla, CA, USA) and anti-CD11b (BioLegend®, San Diego, CA, USA) antibodies.

### 2.2. Transferon® Samples

Three different batches of Transferon® were employed in this work: 16F18, 16G22, and 16H23. Transferon® batches were manufactured and provided by Pharma-FT at ENCB-IPN facilities (Mexico City, Mexico).

### 2.3. Treatments

PMA-differentiated THP-1 cells were treated with Transferon® (0.1, 1.0, or 10.0 *μ*g/mL) in the presence or absence of LPS (Sigma®). The employed concentrations of LPS were 0.1 and 1.0 *μ*g/mL.

### 2.4. CD14, CD11b, CD80, and CD86 Detection by Flow Cytometry

CD14 and CD11b expressions were evaluated using anti-CD14-Pacific Blue (BD Biosciences®, clone M4E2), and anti-CD11b (BioLegend®, clone M1/70) antibodies were used to confirm the PMA-induced differentiation of THP-1 to macrophage-like cells. Macrophage activation markers were analyzed using *α*CD80-PE (BD Biosciences®, clone L307.4), *α*CD86-APC (BD Biosciences®, clone 2331), or *α*HLA-DR-PE antibodies (BD Biosciences®, cloneG46-6). In all cases, cells were incubated with 100 *μ*L of blockade buffer (BD Biosciences®) during 20 min and washed with stain buffer. Subsequently, cells were stained with the corresponding antibodies during 30 min on ice. Finally, cells were washed twice with stain buffer and acquired in a BD FACSAria III cytometer (BD Biosciences®). Data were analyzed using FlowJo® software version 7.6.2. (La Jolla, CA).

### 2.5. Cytokine Detection by Flow Cytometry

Cells were incubated with Brefeldin A (Sigma®) for intracellular detection of IL-1*β*, TNF-*α*, and IL-6. After treatment, cells were washed with 100 *μ*L of Perm/Wash® buffer (BioLegend®) and incubated with 200 *μ*L of Cytofix/Cytoperm™ buffer (BD Biosciences®, 555028) in a light-protected ice bath. After 20 min, cells were washed with stain buffer and the respective antibody was added: anti-TNF-PerCP/Cy5.5 (BioLegend®, clone Mab11), anti-IL-1*β*-Pacific Blue (BioLegend®, clone H1b-98), or anti-IL-6-PE (BioLegend®, clone MQ2-13A5). Then, cells were washed again, suspended in stain buffer, and acquired on a BD FACSAria III cytometer. Data were analyzed using FlowJo software version 7.6.2.

### 2.6. Cytokine Quantitation

IL-1*β*, TNF-*α*, and IL-6 were quantified in supernatants of macrophage-like THP-1 cells treated with LPS and/or Transferon® during 24 and 48 h. IL-1*β* was analyzed using ELISA kit OptEIA Human IL-1*β* (BD Biosciences, 557953). TNF-*α* and IL-6 were analyzed using a human Cytometric Bead Array (CBA) (BD Biosciences®, 560484). Both assays were performed according to the instructions of the manufacturer.

### 2.7. Statistical Analysis

Statistical differences among treatments were determined as follows: the D'Agostino-Pearson omnibus normality test was performed to determine the normal distribution in our study groups, and then a one-way ANOVA followed by a Dunn's multiple-comparison test was performed using GraphPad Prism version 6.00 for Windows, (GraphPad Software, La Jolla California USA; https://www.graphpad.com). A *t*-test was applied to distinguish macrophage-like THP-1 cells from undifferentiated cells. A Pearson correlation was executed to determine the association between IL-6 production and CD80 expression. In all cases, mean ± standard deviation (SD) is reported. The statistical significance is described using these signs: ^∗^ *P* ≤ 0.05, ^∗∗^ *P* ≤ 0.01, and ^∗∗∗^ *P* ≤ 0.001.

## 3. Results

### 3.1. Transferon® Alone Did Not Induce Activation in Macrophage-Like THP1 Cells

**Figure 1:**
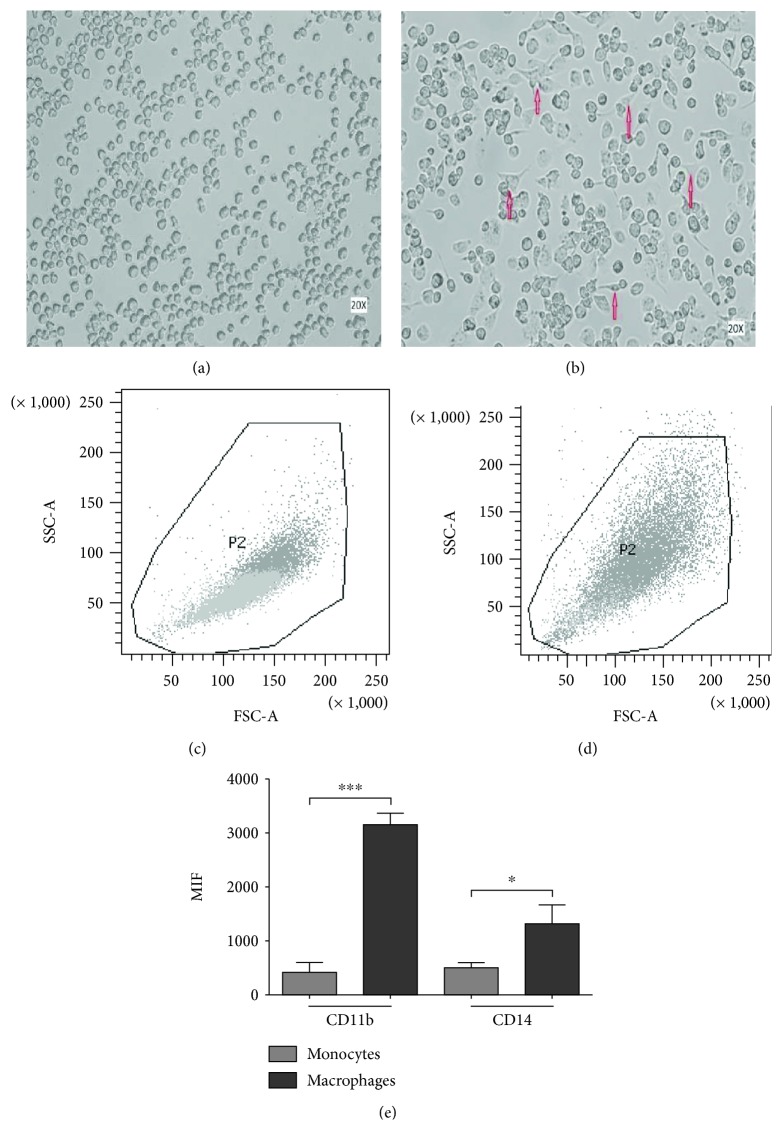
Induction of the macrophage-like phenotype in THP1 cells. THP-1 cells were differentiated by PMA treatment. Morphological changes were analyzed by comparing (a) undifferentiated cells versus (b) differentiated cells. Arrows indicate typical “spreading.” Differentiation was corroborated by analyzing the increment in size and granularity using flow cytometry, comparing (c) nondifferentiated vs. (d) differentiated cells. (e) PMA-treated cells showed increased levels of CD11b and CD14 expression. ^∗∗∗^ *P* ≤ 0.0001.

PMA treatment induced a macrophage-like phenotype in THP-1 cells. After PMA stimulation, the cells showed a typical spreading (Figures 1(a) and 1(b)). Further characterization by flow cytometry confirmed that differentiated cells increased size and granularity (Figures 1(c) and 1(d)) and showed higher levels of CD11b and CD14 on their membrane (Figure 1(e)). The expression levels of both receptors showed significant differences between THP-1 cells before and after differentiation (CD11b: *P* ≤ 0.0001 and CD14: *P* ≤ 0.0001). Only differentiated cells were employed in the subsequent experimental procedures.

**Figure 2:**
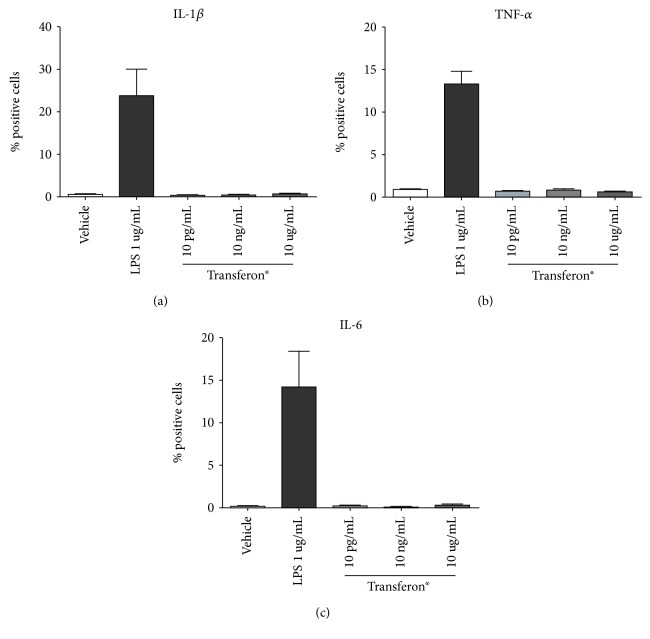
Transferon® alone did not induce cytokine production in macrophage-like THP1 cells. Cells were stimulated with 10 pg/mL, 10 ng/mL, and 10 *μ*g/mL of Transferon®. LPS (1 *μ*g/mL) was used as positive control. Cells were stimulated, and then, Brefeldin A was added. Graphs show the percentage of TNF-*α*-positive cells (a), IL-1*β*-positive cells (b), or IL-6-positive cells (c).

We stimulated THP-1-like macrophages with different concentrations of Transferon® without LPS. In these assays, we did not observe morphological changes nor alterations of the production of IL-1*β*, TNF-*α*, or IL-6 in macrophage-like THP1 cells (Figure 2). This result agrees with the null immunogenicity previously reported for Transferon® [24]. In contrast, stimulation with LPS (1 *μ*g/mL) significantly increased the fraction of positive cells, as expected [25]; treatment with LPS was used as positive control in these experiments.

### 3.2. Transferon® Upregulated IL-6 Production in the Presence of LPS

**Figure 3:**
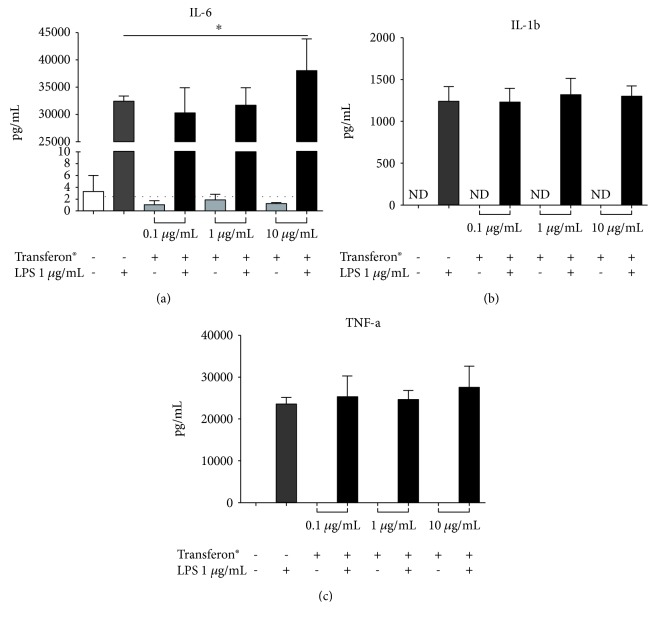
Transferon® increased IL-6 production. Macrophage-like THP1 cells were treated with 0.1 *μ*g/mL, 1 *μ*g/mL, and 10 *μ*g/mL of Transferon® in the presence or absence of 1 mg/mL LPS. Supernatants were collected at 24 h, and (a) IL-6, (b) IL-1*β*, and (c) TNF-*α* quantification was performed by ELISA. ^∗^ *P* ≤ 0.0017.

In the second set of experiments, we tested whether Transferon® could modulate LPS-induced cytokine production. Interestingly, in the simultaneous treatment of macrophage-like THP-1 cells with Transferon® and LPS, IL-6 production was upregulated at high concentrations of Transferon®, which was statistically different when compared with LPS control (*F* = 6.315, *dƒ* = 3, 32; *P* ≤ 0.002) (Figure 3(a)) but it did not affect IL-1*β* and TNF-*α* production (Figures 3(b) and 3(c)).

### 3.3. Transferon® Increased LPS-Induced CD80/CD86 Cell-Surface Expression

**Figure 4:**
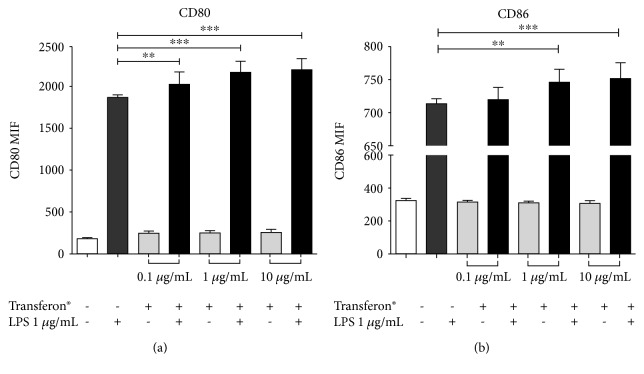
Transferon® increased the expression of costimulatory CD80/CD86 molecules. Macrophage-like THP1 cells were treated with 0.1 *μ*g/mL, 1 *μ*g/mL, and 10 *μ*g/mL of Transferon® with or without LPS. After 24 hours of exposure, the cells were stained with (a) anti-CD80 or (b) anti-CD86. ^∗∗^ *P* < 0.003, ^∗∗∗^ *P* < 0.001.

Macrophage-like THP1 cells were treated with LPS (1 *μ*g/mL) and different concentrations of Transferon® (0.1, 1, and 10 *μ*g/mL) to evaluate the effect of Transferon® on cell surface expression of costimulatory molecules. We observed that CD86 was upregulated in cells simultaneously treated with LPS and Transferon® (1 and 10 *μ*g/mL) in comparison with cells treated with LPS alone (*F* = 9.458, *dƒ* = 3, 32; *P* ≤ 0.0001 for LPS vs. LPS+Transferon® 1 *μ*g/mL and *P* ≤ 0.02 for LPS vs. LPS+Transferon® 10 *μ*g/mL), as shown in Figure 4(a). A similar effect was determined for CD80 expression in all Transferon® concentrations used when compared to LPS control (*F* = 17.22, *dƒ* = 3, 32; Transferon® 0.1 *μ*g/mL *P* ≤ 0.003, Transferon® 1 *μ*g/mL *P* ≤ 0.0001 and Transferon® 10 *μ*g/mL *P* ≤ 0.0001), as shown in Figure 4(b); also, we evaluated HLA-DR surface expression; however, no changes in each condition were observed (see Supplementary Materials (available here)). These results demonstrated that Transferon® has a costimulatory effect on macrophage-like THP1 cells, which could impact on immune response mediated by macrophages.

### 3.4. CD80 Expression and IL-6 Production Showed a Positive Correlation in Cells Costimulated with LPS Plus Transferon®

**Figure 5:**
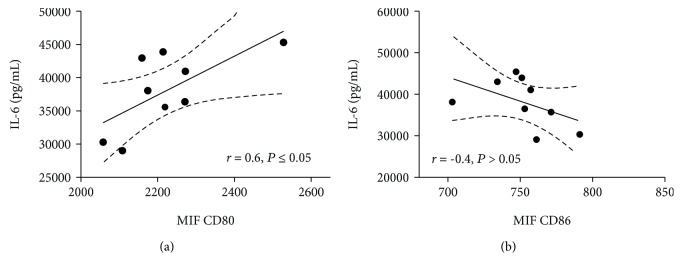
Positive correlation between IL-6 and CD80 expression in cells costimulated with Transferon® and LPS. Linear regression of CD80 and IL-6 (a) or (b) CD86 and IL-6.

An association between CD80 and CD86 expression with IL-6 production in dendritic cells has been recently reported [26]. For this reason, we determined the association between the increment of IL-6 production and changes in CD80/CD86 surface expression in cells treated with Transferon® (10 *μ*g/mL) plus LPS. A Pearson correlation test found a positive correlation between CD80 expression and IL-6 production (*r* = 0.6, *P* ≤ 0.05; Figure 5(a)). However, no correlation for CD86 expression and IL-6 production was observed (Figure 5(b)).

## 4. Discussion

Contradictory effects of DLE have been reported using *in vitro* assays. Our results agree with those reported by Ojeda et al. using a DLE extract made in Cuba [27]. In both reports, DLE increases IL-6 production in HUVECs and primary cultured human monocytes in a similar way to our THP-1 macrophage model, despite using different DLE concentrations and cellular models. In contrast, Franco-Molina et al. reported that a bovine DLE decreases IL-6, TNF-*α*, and IL-1*β* expression [28]; these differences could be attributed to the biological source of DLE, type of sample, and experimental conditions. THP-1 is a human leukemia-derived monocytic cell line that resembles monocytes and macrophages after PMA activation. Accordingly, it has been widely used for *in vitro* studies [29–31]. THP-1 cells can be differentiated to macrophages using PMA. The resulting cells have phenotypic and functional characteristics that resemble those of primary human macrophages [32], including CD11b and CD14 expression. CD11b is an important integrin for the linkage to the extracellular matrix [33], while CD14 functions as coreceptor of TLR4 for LPS recognition [34].

In addition to their key functions in innate response, macrophages play a relevant role in adaptive immune response acting as APCs that load peptides into the MHC-II molecule, allowing interaction with specific T-cell receptors (TCRs). However, T-cell activation requires that the costimulatory receptors CD80 and CD86 bind to CD28 or CD152 on a T-cell surface to complete immunological synapse [35]. The costimulatory receptors drive the activation of T-cell by ligation with CD28 or restrict T-cell proliferation by CD152 ligation [36]. In addition, those interactions cooperate with local cytokine production to define the Th profile. For example, it is well known that IL-2 drives Th1 profile, while IL-4 drives anti-inflammatory Th2 profile [37].

Recently, it has been reported that IL-6 also drives to Th2 profile [18, 38]. IL-6 is a pleiotropic cytokine involved in both pro- and anti-inflammatory processes [39]. As an immunomodulatory cytokine, IL-6 has been related to tissue remodeling and macrophage polarization to M2 profile [40, 41].

Here, we found a positive correlation between IL-6 production and CD80 expression in LPS-activated THP-1-like macrophages in costimulation with Transferon®. Recently, it has been reported that ligation of CD80/CD86 in dendritic cells induces Notch signaling activation and IL-6 production to enhance T-cell response [26]. The binding site of CD80 comprises residues 87-94, which interacts with residues 110 to 120 of CD28 [42]. Since our experimental system did not include T-cells, we hypothesize that some of the low molecular weight peptides present in Transferon® may have a direct binding to CD80, eliciting an enhancement in IL-6 production. IL-6 stimulation could, in turn, induce a positive feedback that increases the expression of CD80 and CD86, as previously reported for dendritic cells [43]. However, further studies are required to determine (i) whether the CD80-activated Notch signaling is active in macrophage-like cells costimulated with Transferon®, (ii) what is the role of such pathway in the increment of IL-6 production, and (iii) what are the functional consequences of this phenomenon.

## 5. Conclusion

Transferon® has been used as an immunomodulatory drug in clinical trials of diseases with an inflammatory component. Due to its complex composition, it is possible that its therapeutic effects are elicited by several different mechanisms in the immune system. Thus, it is important to evaluate the response of different cell lineages to this immunomodulator. Our results showed that Transferon® is able to increase the expression of costimulatory molecules CD80 and CD86 in THP-1 macrophage-like cells. We also identified a new possible mechanism of action that involves CD80 and the production of IL-6.

## Data Availability

The data used to support the findings of this study are available from the corresponding author upon request.

## Supplementary Materials

Supplementary Materials Supplementary Figure 1: Transferon® did not modify HLA-DR expression. Macrophage-like THP1 cells were treated with 0.1 *μ*g/mL, 1 *μ*g/mL, and 10 *μ*g/mL of Transferon® with or without LPS. After 24 hours of exposure, the cells were stained with an anti-HLA-DR.
