# Supplementary material for: CD80 Expression Correlates with IL-6 Production in THP-1-Like Macrophages Costimulated with LPS and Dialyzable Leukocyte Extract (Transferon®)
Source: J Immunol Res. 2019 Apr 10;2019:2198508. doi: 10.1155/2019/2198508 (PMC6481127; doi:10.1155/2019/2198508)
Supplement: Supplementary Materials — Supplementary Figure 1: Transferon® did not modify HLA-DR expression. Macrophage-like THP1 cells were treated with 0.1 μg/mL, 1 μg/mL, and 10 μg/mL of Transferon® with or without LPS. After 24 hours of exposure, the cells were stained with an anti-HLA-DR. [file 2198508.f1.docx]

**CD80 expression correlates with IL-6 production in THP-1 like macrophages costimulated with LPS and Dialyzable Leukocyte Extract (Transferon^®^)**

Alexis P. Jiménez-Uribe^§1^, Hugo Valencia-Martínez^§1^, Gregorio Carballo-Uicab^1^, Luis Vallejo-Castillo^1,2^, Emilio Medina-Rivero^1^, Rommel Chacón-Salinas^1,3^, Lenin Pavón*^4^, Marco A. Velasco-Velázquez^5^, Gabriela Mellado-Sánchez^1^, Sergio Estrada-Parra^3^, Sonia M. Pérez-Tapia*^1,3^.

^1^ Unidad de Desarrollo e Investigación en Bioprocesos (UDIBI), Escuela Nacional de Ciencias Biológicas, Instituto Politécnico Nacional. Ciudad de México 11340, México.

^2^ Departamento de Farmacología, Centro de Investigación y de Estudios Avanzados (Cinvestav) del IPN. Ciudad de México 07360, México.

^3^ Departamento de Inmunología, Escuela Nacional de Ciencias Biológicas, Instituto Politécnico Nacional. Ciudad de México 11340, México.

^4^ Laboratorio de Psicoinmunología, Dirección de Investigaciones en Neurociencias del Instituto Nacional de Psiquiatría Ramón de la Fuente. Ciudad de México 14370, México.

^5^ Departamento de Farmacología y Unidad Periférica de Investigación en Biomedicina Traslacional (CMN 20 de noviembre, ISSSTE), Facultad de Medicina, Universidad Nacional Autónoma de México, Ciudad Universitaria. Ciudad de México 04510, México.


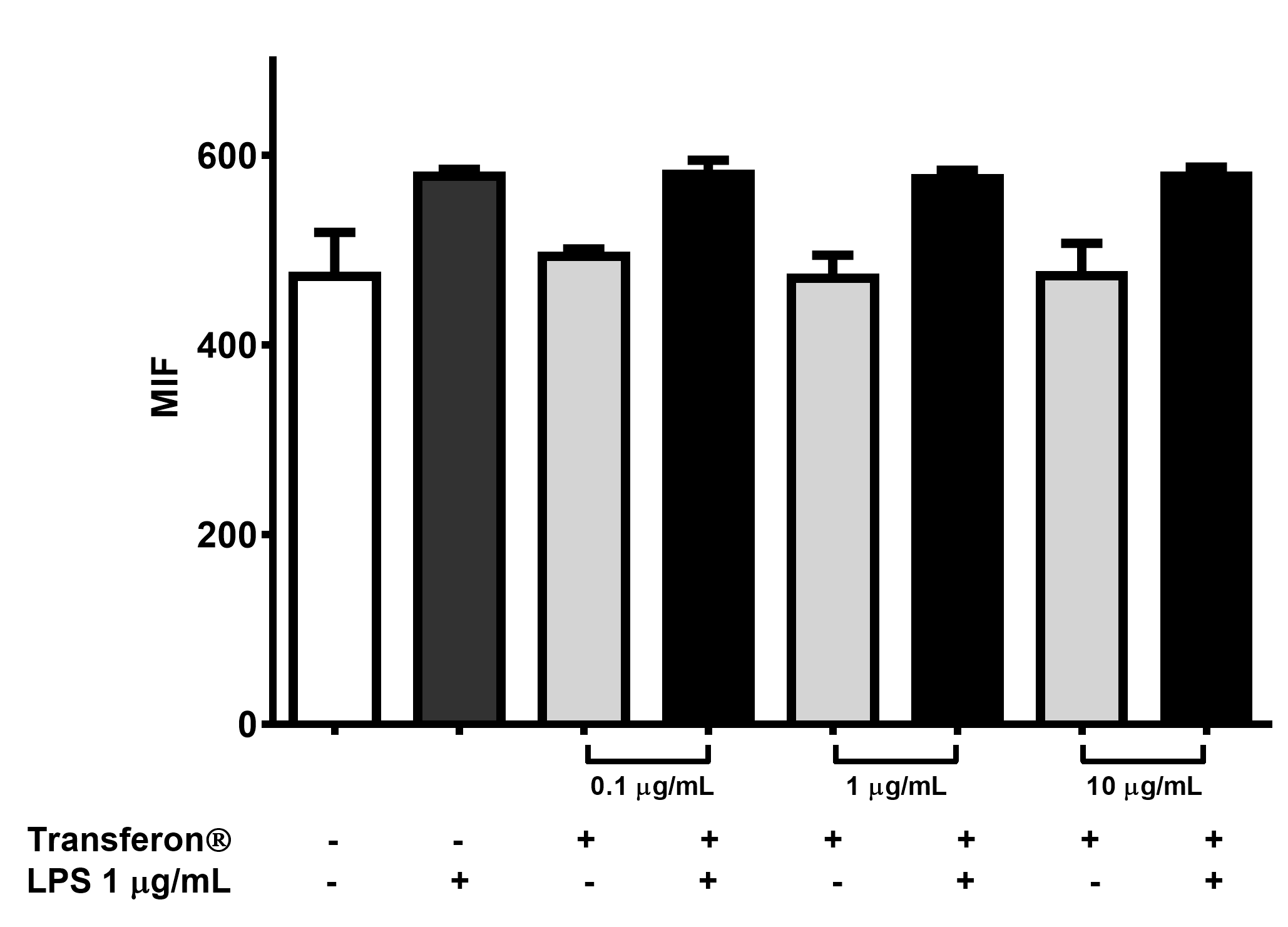


**Supplementary Figure 1. Transferon^®^ did not modified HLA-DR expression** Macrophage-like THP1 cells were treated with 0.1 μg/mL, 1 μg/mL and 10 μg/mL of Transferon^®^ with or without LPS. After 24 hours of exposure, the cells were stained with an anti-HLA-DR.
